# Obstetric emergency simulation training course: experience of a private-public partnership in Brazil

**DOI:** 10.1186/s12978-019-0689-6

**Published:** 2019-02-27

**Authors:** Monica Maria Siaulys, Lissandra Borba da Cunha, Maria Regina Torloni, Mario Macoto Kondo

**Affiliations:** 1 Hospital e Maternidade Santa Joana, Centro de Ensino, Pesquisa e Inovação, Rua Dr. Eduardo Amaro 225, São Paulo, SP CEP 04104 080 Brazil; 2 Evidence Based Healthcare Post graduate Programme, Department of Medicine, São Paulo Federal University, Rua Botucatu 740, 3o andar, São Paulo, SP CEP 04023-900 Brazil

**Keywords:** Simulation training, Obstetrics, Public-private sector partnerships, Clinical competence, Eclampsia, Postpartum hemorrhage, Sepsis, Cardiopulmonary resuscitation, Brazil, Treinamento por simulação, Obstetrícia, Parcerias Público-Privadas, Competência Clínica, Eclampsia, Hemorragia Pós-Parto, Sepse, Reanimação Cardiopulmonar, Brasil

## Abstract

**Background:**

Lack of skills on how to diagnose and manage obstetric emergencies contribute to substandard institutional care and preventable maternal deaths in Brazil. Simulation-based obstetric emergency team training can reduce adverse maternal outcomes. However, this type of training is expensive and not widely available, especially in low resource settings. We present the experience of a private-public partnership that offered a two-day obstetric emergency simulation-training course to hundreds of Brazilian professionals working in the public sector. We also present participants´ short-term learning outcomes (Kirkpatrick’s level 2) and satisfaction (Kirkpatrick’s level 1).

**Methods:**

This was a non-experimental before-and-after study. The free 16-h course was held over a 14 months period in a large private hospital’s simulation center using multidisciplinary scenario and model-based training. The training sessions consisted of four (4-h) modules on pre-eclampsia/eclampsia, hemorrhage, sepsis and resuscitation. An anonymous questionnaire collected participants´ satisfaction at the end of each module. Learning outcomes were assessed by comparing differences in participants´ pre- versus immediate post-course test scores. Wilcoxon, Kruskal-Wallis and Friedman tests were used for statistical analyses. *P* < 0.05 was considered significant.

**Results:**

340 professionals (117 doctors, 179 registered nurses-RN and 44 licensed practical nurses-LPN) working in 33 public Brazilian hospitals were trained. There was a significant increase in post-course test scores in all four modules. On average, scores increased 55% in the hypertension and 65–69% in the hemorrhage, sepsis and resuscitation modules (*p* = 0.019). Knowledge acquisition of RN and LPN was similar in the hypertension, hemorrhage and sepsis modules and significantly higher than doctors´ (*p* < 0.05). On a 0 to 10 scale, mean overall satisfaction ranged from 9.6 (for the hypertension module) to 9.8 (for the resuscitation module).

**Conclusions:**

This successful experience of a private-public partnership to offer obstetric emergency simulation training required strategic organization and a strong commitment from both sides. This promising private-public partnership model could be replicated in similar settings. The training course obtained high satisfaction scores and significantly improved the knowledge of public-sector health professionals on how to manage the main causes of maternal mortality.

## Resumo

**Introdução:**

Deficiências na capacidade de diagnosticar e tratar emergências obstétricas contribuem para um tratamento hospitalar de menor qualidade e mortes maternas evitáveis no Brasil. O treinamento por simulação de equipes para atender emergências obstétricas pode reduzir desfechos maternos adversos. Porém, esse tipo de treinamento não é disponível em larga escala, especialmente em locais com poucos recursos. Apresentamos a experiência de uma parceria privado-pública que ofereceu um treinamento por simulação de dois dias sobre emergências obstétricas para centenas de profissionais de saúde brasileiros que trabalhavam no setor público. Também apresentamos a melhora dos conhecimentos dos participantes no curto prazo (nível 2 de Kirkpatrick) e sua satisfação (nível 1 de Kirkpatrick).

**Métodos:**

Este foi um estudo não experimental do tipo antes-e-depois. O treinamento gratuito, com duração total de 16 horas, foi oferecido ao longo de 14 meses no centro de simulação de um grande hospital privado, usando cenários multidisciplinares e modelos. O treinamento consistia de quatro módulos (4 horas cada) sobre pré-eclampsia/eclampsia, hemorragia, sepse e reanimação. Ao final de cada módulo, os participantes preencheram um questionário anônimo para avaliar sua satisfação. A aprendizagem foi avaliada comparando as notas dos participantes nos testes realizados antes e depois de cada módulo. Usamos os testes de Wilcoxon, Kruskal-Wallis e Friedman para as análises estatísticas. Valores de *P* < 0,05 foram considerados significativos.

**Resultados:**

340 profissionais de saúde (117 médicos, 179 enfermeiras e 44 técnicas de enfermagem) que trabalhavam em 33 hospitais públicos brasileiros foram treinados. Houve um aumento significativo nos escores dos testes realizados após cada módulo. O aumento médio dos escores foi de 55% no módulo de hipertensão e de 65–69% nos módulos de hemorragia, sepse e reanimação (*p* = 0,019). A aprendizagem das enfermeiras e técnicas de enfermagem foi semelhante nos módulos de hipertensão, hemorragia e sepse, e significativamente maior do que a dos médicos (*p* < 0,05). A satisfação média dos participantes, numa escala de 0 a 10, variou de 9,6 (módulo de hipertensão) até 9,8 (módulo de reanimação).

**Conclusões:**

Essa exitosa experiência de uma parceria privado-pública para oferecer treinamento em emergências obstétricas usando simulação exigiu organização estratégica e um firme empenho de ambos os parceiros. Esse modelo promissor de parceria privado-pública pode ser repetido em locais semelhantes. O treinamento teve notas de satisfação elevadas e aumentou de forma significativa o conhecimento de profissionais de saúde do setor público sobre como conduzir os casos responsáveis pela maioria das mortes maternas.

## Plain language summary

Many doctors and nurses lack skills on how to manage emergencies that can affect pregnant women. This contributes to poor hospital care and preventable maternal deaths. Simulation-based obstetric emergency training can help to improve this problem; but this type of training is expensive and not widely available. We present the experience and results of a private-public partnership that offered a two-day obstetric emergency simulation-training course to hundreds of Brazilian doctors and nurses who worked in public hospitals.

The free course was held in a large private hospital’s simulation center. Each student went through four (4-h) training sessions (on pre-eclampsia/eclampsia, hemorrhage, sepsis and resuscitation). We assessed their satisfaction and measured how much they learned at the end of each module.

Over 14 months, we trained 340 professionals (117 doctors, 223 nurses) who worked in 33 public Brazilian hospitals. Most were highly satisfied with the course; there was a significant increase in post-course test scores in all four modules. On average, test scores increased 55% in the hypertension and 65–69% in the hemorrhage, sepsis and resuscitation modules. We discuss the main obstacles and ingredients for the success of this type of partnership between private and public hospitals.

This promising private-public partnership model could be replicated in similar settings, to improve the quality of care offered to pregnant women in developing countries.

## Background

Brazilian women have a 1/1200 risk of dying during their lives due to maternal causes. This is twice the risk of women in Chile or Russia and ten times the risk of women in Japan or Switzerland [1]. Although Brazil’s national maternal mortality ratio (MMR) is decreasing, current levels (44/100,000 livebirths) and the large differences when compared to other middle-income countries are unacceptable [1]. Over 95% of all births in Brazil occur in hospital facilities and are attended by skilled health personnel [2]. Yet, the major direct causes of maternal death in the country are still hypertensive disorders of pregnancy, hemorrhage, and sepsis [3]. In Brazil, as in many middle-income countries, the main challenge is not access to health services nor lack of knowledge about affordable and effective evidence-based interventions to prevent maternal deaths, but rather the capacity to deliver key interventions in a timely and appropriate manner to those who need it, when they need it [4].

Lack of skills and knowledge on how to recognize and manage obstetric emergencies contribute to substandard institutional care and preventable maternal deaths [5]. While most agree that the acquisition of skills and continuous training are important, not all training packages for obstetric emergencies are equal or effective. High-fidelity simulation-based multidisciplinary training may yield better results than other types of training [6–8]. Simulation-based obstetric emergency team training can improve participants´ knowledge and skills, and increase their ability to detect and appropriately manage relatively rare life-threatening events that can affect women during pregnancy, labor and the post-partum period [6, 9, 10]. This type of training can have a positive impact on the aptitudes of course participants, and consequently on patients and organizations [6, 9–13]. However, simulation-based emergency obstetric courses require trained teachers, infrastructure, devices and life-like virtual environments to mimic events that occur in clinical encounters. The initial financial investments required may represent a barrier to the widespread use of this type of training, especially in low resource settings [14–16].

The World Health Organization (WHO) reproductive health strategy to accelerate progress towards the attainment of international development goals and targets advocates for collaboration and complementary action among the private, non-governmental and public sectors to maximize availability and use of reproductive health services [17]. Therefore, a possible solution to the financial challenge of obstetrics emergency training is a partnership between a private simulation center and public hospitals to offer this high quality training to a large number of healthcare providers who work in the public sector. To the best of our knowledge, this model of partnership for training healthcare professionals has not been described in low and middle-income countries.

There are several ways to assess if the objectives of a training program were met. The Kirkpatrick four-level framework [18], one of the most used in studies involving training of healthcare professionals, proposes to assess participants´ reaction (satisfaction) with the learning experience (level 1), changes in their knowledge or skills (level 2), changes in their behavior in a clinical setting (level 3) and changes in organizational (patient) outcomes (level 4).

## Methods

The main objectives of this study were to present the experience and short-term learning outcomes of a private-public partnership in Sao Paulo, Brazil, to train public health sector personnel on the management of obstetric emergencies using a high-fidelity, simulation-based, multiprofessional course. This non-experimental before-and-after study took place in Sao Paulo, Brazil, between 2016 and 2017.

The Santa Joana Group is a private institution that owns five for-profit hospitals in Sao Paulo and Rio de Janeiro. Each year, these hospitals deliver together a total of approximately 44,000 women. All clinical staff of the Santa Joana Group, including obstetricians, anesthesiologists, registered nurses (RN) and licensed practical nurses (LPN), go through annual mandatory in-service trainings on how to manage pre-eclampsia/eclampsia, hemorrhage, sepsis and basic life support (BLS) procedures for maternal and neonatal arrest. Prior to 2014, this training involved didactic lectures, written handouts and drills using low fidelity mannequins. In 2014, the Santa Joana Group built a simulation center in the Santa Joana Maternity Hospital (in Sao Paulo city) and hired a dedicated team of instructors accredited to teach multidisciplinary scenario and model based training courses.

In early 2016, in an effort to help reduce the number of preventable maternal deaths in the state of Sao Paulo, the Santa Joana Group contacted authorities of the state health department and offered to train, in its simulation center, healthcare professionals working in public maternities. A private-public partnership agreement was formally established between the two institutions. The objective of this partnership was to revise and improve obstetric emergency skills of healthcare workers managing deliveries in public maternity hospitals in the state of Sao Paulo. The training was provided free of charge. The Sao Paulo state health department allowed healthcare professionals working in public maternities to leave their activities for two days to participate in the training course in Sao Paulo city and covered their lodging and food costs. The state health department selected the order in which each of the state’s public maternities would be included in the training process, based mainly on the historical MMR of the regions served by these institutions. The public maternity directors selected the number, type of professional, and order of its staff members who would participate in the simulation course.

The training sessions consisted of four (4-h) modules conducted by dedicated multidisciplinary instructors (two anesthesiologists and two obstetric RNs) from the Santa Joana Group, all with special education in leading simulation training and certified by Laerdal [19]. The content and format of the sessions were based on the Practical Obstetric Multi-Professional Training (PROMPT) training course manual [20]. This multidisciplinary training program was originally developed in the UK in 1998 to improve the outcomes for British mothers and their babies during obstetric emergencies [21]. This program was tested in randomized trials and shown to be effective in high-income countries [22] and to reduce maternal mortality in Africa [23]. The four modules (pre-eclampsia/eclampsia, hemorrhage, sepsis and BLS) were taught over a two-day period (total course duration: 16 h). Prior to each module, participants took a written multiple-choice questions (MCQ) test to assess their pre-course knowledge about the topic of the upcoming module. The test questions were created by the course instructors, based on the module content and previous studies [9, 10, 24]. The participants then listened to a short lecture about the importance of the module’s topic as a cause for maternal mortality in Brazil, and evidence-based diagnosis and management of the condition. The participants then split into two smaller (five persons) multiprofessional groups. Each group went into separate training rooms and practiced, under the direct supervision of an instructor, how to diagnose and manage the condition using real equipment (e.g. MgSO4 ampoules and syringes) and low fidelity mannequins (Resusci Anne Simulator, Automatic External Defibrillation Trainer and Multi-Venous IV Training Arm, Laerdal Medical, Stavanger, Norway). All students and instructors then reconvened for a group discussion about the main diagnostic and management steps of the disorder. After a short break, participants formed two smaller multiprofessional teams (RN, PLN and physicians). The first team went into a room with a computer-controlled high fidelity mannequin (SimMom, Laerdal Medical, Stavanger, Norway) that responded to treatment. These students practiced the management of a simulated obstetric emergency, without the presence of an instructor, to test their competency as a team in managing the patient. This training was videotaped and broadcast live to the second team of students in the lecture room. The second team of students and an instructor observed the first team on a TV screen and assessed their teamwork using a standardized questionnaire. At the end of the session, all participants and instructors reconvened, watched the video recording of the first team’s performance and debriefed. At the end of the module, all participants took again a written MCQ test; the questions were similar to the first test and in a different order. Finally, all participants completed an anonymous questionnaire using a four-points scale (bad, regular, good, excellent) to assess their reaction to the module (methods, expectations, instructors and resources). After lunch, participants reconvened in the lecture room to start the second module, which followed the same sequence as the first. The third and fourth modules were similar to the first two, and occurred on the second day of the training course.

All course participants provided written informed consent at enrollment and the study was approved by the Santa Joana Group institutional review board. We assessed participants´ reaction (Kirkpatrick level 1) [18] to each of the four course modules based on their answers to the satisfaction questionnaires. We assessed participants´ short-term knowledge acquisition (Kirkpatrick level 2) [18] by calculating the change in their test scores using the formula: [(post-course score - pre-course score)/ pre-course score] × 100. We compared differences in score changes in each of the four modules (pre-eclampsia/eclampsia, hemorrhage, sepsis and BLS) and between physicians, RN and LPN. Participants who did not complete both the pre and post-course knowledge questionnaires were excluded from knowledge acquisition analyses. We used R software (R Core Team, version 2.12.0, Vienna, Austria) and the Wilcoxon, Kruskal-Wallis and Friedman tests for statistical analyses. *P* < 0.05 was considered significant.

## Results

Between October 2016 and December 2017, the same team of instructors trained 365 healthcare professionals distributed over 32 editions of two-day courses. We present the results of the 340 (93.1%) participants who completed both the pre- and post-course tests.

Participants worked in 33 different public secondary and tertiary hospitals in the state of Sao Paulo and two thirds (*n* = 223, 65.6%) were nursing professionals (Table 1). Most of the teams of participants consisted of professionals that worked together in the same department and institutions. Among the 117 physicians, there were 19 obstetrics/gynecology residents, in the 2nd or 3rd years of their programs. Nearly 80% of the participants (*n* = 264) attended all four modules. Over 300 professionals participated in each of the individual modules (Table 1).

**Table 1:** Characteristics of 340 participants in emergency obstetric training course, São Paulo, 2016–2017

Characteristics	N	%
Types of participants		
Doctors	117	34.4
Registered nurses	179	52.6
Licensed practical nurses	44	13.0
Modules completed by participants		
4 modules	264	77.6
3 modules	49	14.4
2 modules	20	5.9
1 module	7	2.1
Participants per modude		
Hypertension	322	94.7
Resuscitation	318	93.5
Sepsis	308	90.6
Hemorrhage	302	88.8

Mean (standard deviation) pre-course scores of the 340 participants were lowest for the sepsis (5.9 + 1.7, maximum score of 10) and highest for the hypertension (6.3 + 1.8) modules, but differences did not reach statistical significance (*p* = 0.051) (Table 2). There was a significant increase in post-course test scores in all four modules (*p* < 0.001). On average, scores increased 55% in the hypertension and 65–69% in the hemorrhage, sepsis and resuscitation modules. The average proportional change in scores ([(post-course score - pre-course score)/ pre-course score] × 100) was similar in the hemorrhage, sepsis and resuscitation modules (*p* > 0.05) and significantly higher than in the hypertension module, *p* = 0.019). In all modules, over 90% of the participants had an increase in their post-course scores. (Table 2).

**Table 2:** Knowledge acquisition of 340 health professionals participating in emergency obstetric training course, São Paulo, 2016–2017

	Hypertension	Hemorrhage	Sepsis	Resuscitation
	(*N* = 322)	(*N* = 302)	(*N* = 308)	(*N* = 318)
Mean pre-course score (SD)	6.3 (1.8)^a^	6.0 (1.9)^a^	5.9 (1.7)^a^	6.1 (2.0)^a^
Mean post-course score (SD)	8.8 (1.1)^b^	8.9 (1.1)^b^	8.8 (1.1)^b^	9.2 (1.0)^b^
Mean percent score change^d^	54.8^c^	68.6^c^	65.1^c^	69.0^c^
% participants with increased scores	90.1	91.4	92.5	91.8

^a^*p* = 0.051, Friedman test, mean pre-course test scores between modules

^b^*P* < 0.001, Wilcoxon test, pre versus post-course scores

^c^*p* = 0.019, Friedman test, % score change between hypertension versus other modules

^d^Percent score change = ((post-course score - pre-course score)/pre-course score)) × 100

All professional categories (doctors, RN and LPN) had a significant increase in test scores at the end of each of the four modules (*p* < 0.001). There were no significant differences in the knowledge acquisition (mean percent score change) of RN and LPN in the hypertension, hemorrhage and sepsis modules; their knowledge acquisition was significantly higher than doctors´ (*p* < 0.05). In the BLS module, LPN had a significantly higher knowledge acquisition than RN and doctors (p < 0.05) but there were no significant differences between the knowledge acquisition of RN and doctors (Table 3).

**Table 3:** Knowledge acquisition of staff groups in modules of emergency obstetric training course, São Paulo, 2016–2017

	Hypertension	Hemorrhage	Sepsis	Resuscitation
Doctors	(*n* = 112)	(*n* = 102)	(*n* = 102)	(*n* = 108)
Mean pre-course score (SD)	7.5 (1.4)	7.2 (1.6)	6.6 (1.6)	6.5 (1.9)
Mean post-course score (SD)	9.1 (1.0) ^a^	9.3 (0.9)^a^	9.3 (0.9) ^a^	9.4 (0.8) ^a^
Mean % score change	25.7^b^	37.8^b^	49.3^c^	60.4^d^
Registered nurses	(*n* = 168)	(*n* = 160)	(*n* = 162)	(*n* = 173)
Mean pre-course score (SD)	5.9 (1.6)	5.5 (1.7)	5.6 (1.7)	6.1 (2.0)
Mean post-course score (SD)	8.7 (1.1)^a^	8.8 (1.1)^a^	8.7 (1.1)^a^	9.1 (1.0)^a^
Mean % score change	63.6^b^	79.5^b^	70.6^c^	67.3^d^
Licensed practical nurses	(*n* = 42)	(*n* = 40)	(*n* = 44)	(*n* = 37)
Mean pre-course score (SD)	5.3 (1.9)	5.0 (1.8)	5.1 (1.5)	5.0 (1.8)
Mean post-course score (SD)	8.8 (1.2)^a^	8.5 (1.3)^a^	8.3 (1.2)^a^	9.0 (1.1)^a^
Mean % score change	97.1^b^	103.5^b^	81.2^c^	101.4^d^

^a^*P* < 0.001, Wilcoxon test, pre versus post-course scores

^b^*P* < 0.001, Kruskal-Wallis test, score change of registered nurses vs doctors and licensed practical nurses vs doctors

^c^*P* < 0.05, Kruskal-Wallis test, score change between registered nurses vs doctors and licensed practical nurses vs doctors

^d^*p* < 0.05, Kruskal-Wallis test, score change between licensed practical nurses vs registered nurses and licensed practical nurses vs doctors

Percent score change = ((post-course score - pre-course score)/pre-course score)) × 100

The mean (SD) overall satisfaction (0 to 10 scale) of the participants was 9.6 (0.9), 9.8 (0.5), 9.7 (0.6) and 9.8 (0.8) in the hypertension, hemorrhage, sepsis and resuscitation modules, respectively. In each module, over 84% of the participants rated all items assessed in the four domains (methods, expectations, instructor and resources) as ´excellent´. (Table 4). Due to the low number of ´bad and ´regular´ scores, we combined these answers into a single group.

**Table 4:** Reaction of 340 participants to obstetric emergency course modules, São Paulo 2016–2017

	Hypertension module			Hemorrhage module			Sepsis module			Resuscitation module		
	*N* = 298			*N* = 274			N = 308			*N* = 276		
Domains	bad or regular	good	excellent	bad or regular	good	excellent	bad or regular	good	excellent	bad or regular	good	excellent
Methods												
content	1.0	14.6	84.4	0.7	8.8	90.5	0.6	9.0	90.4	0.4	8.0	91.7
strategy to present content	1.4	11.2	87.5	0.7	8.8	90.5	0	8.7	91.3	1.1	6.9	92.0
organization of activities	0.7	10.8	88.5	0	8.4	91.6	0	8.4	91.6	0.4	6.5	93.1
Expectations												
fulfilled my expectations	1.7	13.2	85.1	2.2	10.9	86.9	0.6	10.0	89.4	2.2	6.5	91.3
useful for clinical practice	1.0	8.8	90.2	1.5	8.0	90.5	1.0	5.8	93.2	1.1	6.5	92.4
contributed to professional growth	0.3	7.5	92.2	1.1	4.4	94.5	0	3.9	96.1	0.7	2.9	96.4
Instructors												
knowledge of content	0.3	6.8	92.9	0.7	5.5	93.8	0	4.2	95.8	0.7	6.2	93.1
communication/posture	0.3	5.4	94.3	0	4.7	95.3	0.3	4.2	95.5	0.7	3.3	96.0
response to questions	0.7	5.7	93.6	0.7	4.4	94.9	0.3	6.2	93.5	1.1	3.3	95.7
Resources												
simulation/manequins	0.7	10.5	88.9	1.5	7.4	91.2	0.3	11.0	88.7	1.1	6.2	92.7
room / ambiance	0.7	13.9	85.5	1.8	9.5	88.6	1.0	14.6	84.4	1.5	9.1	89.5

All data expressed in percentages

## Discussion

This successful private-public partnership trained over 360 Brazilian health professionals in emergency obstetric care over one year. The vast majority of participants had high satisfaction scores, i.e. a positive reaction (Kirkpatrick level 1) to the course. Although this was not one of our primary study objectives, and despite some controversies as to the importance of this item in the assessment of training programs, the reaction of trainees cannot be ignored as it can play a role in building interest and enhancing motivation to learn [25]. As expected [26], there was also a significant increase in participants´ knowledge (Kirkpatrick level 2) on how to manage the major causes of maternal mortality and morbidity, with similar results across cadres. Comparable studies conducted with physicians and midwives in several high and middle-income countries using equivalent training interventions reported similar results [6, 11, 12, 22, 24, 27].

This partnership between the private and public sectors to train healthcare professionals in emergency obstetric skills is an innovative approach that can help reduce maternal mortality and morbidity. The private sector has a social responsibility and can contribute towards improving reproductive health [28]. According to the WHO, collaboration and complementary actions between the private, nongovernmental and public health sectors to train and retain skilled health personnel are central elements in improving health care, especially in settings where inadequate human resources are a major barrier to the expansion of comprehensive reproductive and sexual health services [17]. In this case, the partnership was born because of the private hospital director’s sense of social responsibility and initiative. However, public health authorities can initiate this collaboration and replicate this experience in similar settings in Latin America and other low and middle-income countries. To promote the partnerships, governments could establish incentives for these collaborations by creating some type of reward or compensation based on local values and norms. These incentives do not necessarily need to be material or financial. The reward, as in this case, can simply be the satisfaction that a partner gains from satisfying a sense of mission. If properly framed, collaborations can also increase the credibility of the private institution by its association with respected governmental agencies or elevate its social status by disseminating the results of successful projects to internal and external stakeholders [29].

One of the main difficulties encountered in this specific experience was the legal paper work to officiate the partnership between the private donor, who was offering the training course free of charge and without any other form of compensation, and the public health authorities. Since this type of partnership was new, it took several weeks and discussions between the legal departments of the two partners to draft the appropriate documents that were finally approved by both stakeholders. The strong commitment and determination of both the private and public authorities were essential to make this initiative work. On the public sector’s side, this involved the hospital directors arranging for its staff to attend the course during their working hours and to cover their lodging and food costs for the two days of training. From the private sector’s side, this involved being flexible to adjust the days of the training modules to accommodate to the availability of the participants.

Training in multiprofessional teams is not common in Brazil. However, this did not turn out to be a problem because the private hospital trainers had ample experience with this type of training and made doctors, nurses and LPN feel at ease with this type of experience at the onset of each session.

Our study had several strong points, including its originality and the number of participants trained. We identified only one previous study on emergency obstetric training in Brazil, conducted in 2013, which compared two simulation methods to teach residents how to manage post-partum hemorrhage [30, 31]. To the best of our knowledge, this the first study to present the results of a complete obstetric emergency simulation-training course in Brazil, and it is one of the largest studies on this topic in Latin America [12, 16]. It is also the first publication on a private-public partnership for training in obstetric emergency training. The format of our course was based on a recognized and tested model (PROMPT) and involved exposure of the participants to different instructional strategies (lectures, scenarios and debriefing) which is considered a good simulation design. Moreover, all training was done in small groups of healthcare professionals with different backgrounds (physicians, RN and PLN), who were used to working together on the labor ward. Team approach training, which stresses the importance of all team members working and communicating effectively together, reduces errors and improves patient safety and quality of care in obstetric emergencies [24, 32, 33]. Finally, we followed the guidelines proposed for reporting training programs in low resource settings [15].

Our study had several limitations. First, this was a study conducted in single institution, using a before-and-after design, without a comparison group. Second, we did not assess the actual use of the skills in the local hospitals (Kirkpatrick level 3) or the impact of the training course on relevant clinical patient outcomes, such as maternal mortality (Kirkpatrick level 4, [18]). Measuring behavioral change in 365 participants working in over 30 different hospitals would require a reliable and valid assessment tool in Portuguese, which is not yet available. We decided not use proxy measures, such as healthcare professionals´ self-reports or medical record reviews, since there is limited evidence supporting these methods [34]. Since maternal death is a relatively rare event in individual hospitals, it would take several years to measure differences in this outcome after a training course. Another potential limitation of our study was that training was not conducted in the local hospitals (´in situ´) but at a single central simulation center (´off-site´), distant and different from the participants´ usual place of work. Although many investigators consider in situ courses to be ideal, evidence to support this concept is not unequivocal [6]. Qualitative research indicates that healthcare professionals do not think that physical context or fidelity of training are the most important aspects for learning, and most professionals see the ability of working in different places as an important skill [35, 36]. Evidence surrounding the efficacy of in situ versus off-site simulation training is emerging and this is an unresolved issue [37]. There are also cost implications to conducting and maintaining in situ simulation training, since it involves the need to audit the quality of these courses and instructors [27]. Finally, we acknowledge that our results cannot be generalized because course participants were selected by the hospital directors and may represent the professionals most in need of training, and who accepted to participate in the course.

We plan to conduct a follow-up study with our 340 participants to assess knowledge retention. We will also monitor MMRs in the regions of the participating hospitals, over the next five years.

## Conclusions

This successful experience of a private-public partnership to offer high-fidelity simulation training required strategic organization and a strong commitment from both sides. This promising private-public partnership model could be replicated in similar settings in Brazil and other developing countries and potentially improve the quality of care offered to pregnant women admitted to public hospitals. The Santa Joana Group obstetric emergency simulation-training course obtained high satisfaction scores from the participants and significantly improved the short-term knowledge of public-sector health professionals on how to diagnose and manage the main causes of maternal mortality. Future research should evaluate the effects of training on measurable clinical outcomes such as severe maternal morbidity and mortality.

## Abbreviations

BLS: Basic life support
LPN: Licensed practical nurse
MCQ: Multiple-choice questions
MMR: maternal mortality ratio
PROMPT: Practical Obstetric Multi-Professional Training
RN: Registered nurse
WHO: World Health Organization
